# Conjunctival Provocation Test With *Blomia tropicalis*

**DOI:** 10.3389/falgy.2021.673462

**Published:** 2021-05-07

**Authors:** Elizabeth Maria Mercer Mourao, Nelson Augusto Rosario

**Affiliations:** Department of Pediatrics, Federal University of Parana, Curitiba, Brazil

**Keywords:** allergic rhinoconjunctivitis, mite allergy, allergic conjunctivitis, *Blomia tropicalis*, conjunctival provocation test

## Abstract

**Background:** Conjunctival provocation test (CPT) is used to demonstrate clinical relevance to a specific allergen. *Blomia tropicalis* (Bt) is a prevalent allergen in tropical regions. Its major allergen *Blo t 5* is commonly detected in house dust in Brazil. Patients with allergic rhinoconjunctivitis (ARC) have IgE antibodies to Bt although it may not indicate clinical allergy.

**Objective:** The purpose of this study is to demonstrate the role of CPT in clinical allergy to Bt in allergic conjunctivitis (AC).

**Methods:** CPT was performed in asymptomatic subjects with ARC (*n* = 26) outside the grass pollen season. They had positive skin prick tests (SPT) to Bt and other common inhalant allergens and they were off topical or systemic antihistamines. Standardized allergens were used for CPT (Blo t 5 462.5 ng/mL in 1:1 solution, Alk Abelló). CPT was conducted on a control group of subjects (*n* = 29) without symptoms of ARC and with negative SPT. CPT was performed with progressive doses of allergen solutions in normal saline (1:32, 1:16, 1:8, 1:4, 1:2). CPT with the same allergen dose that elicited a positive reaction was repeated one week later. The protocol was approved by the local Ethics Board and signed informed consent was obtained from all participants.

**Results:** There were 92% (24/26) of positive CPT in subjects sensitized to Bt. Significant association was found between SPT and CPT results with Bt (*p* < 0.0001). CPT had 92% sensitivity and 100% specificity when compared to SPT results. Positive reactions with the same dose or one immediately higher occurred in 21 out of 22 subjects who repeated TPC 1 week later. Mild transient nasal symptoms (21/24) were the major side effects of positive CPT followed by moderate periorbital edema which occurred in 41% (10/24). One controlled asthmatic BT-sensitized subject developed wheezing and dyspnea during a positive CPT with Bt that cleared with inhaled albuterol (400 mcg). There were no reactions whatsoever of CPT in non-allergic subjects.

**Conclusion:** This study demonstrated that Bt may cause allergic conjunctivitis in our population. In addition, CPT is a safe and reproducible test if standardized allergens are used.

## Introduction

*Blomia tropicalis* (Bt) is a common source of mite allergen sensitization in tropical and subtropical countries causing allergic respiratory diseases such as asthma and allergic rhinoconjunctivitis (ARC). The high temperatures and high humidity levels favor mite growth throughout the year leading to early sensitization and persistent symptoms (1). An increase of ARC prevalence has been observed over the last decades in the Tropics and in Brazil when compared to temperate climate regions, with significant impairment of quality of life, mostly affecting older children (2). Originally classified in the 1970s as a storage mite present in stocked grains, Bt is now recognized as an important indoor allergen. It often coexists with *Dermatophagoides pteronyssinus* (Dp) in house dust samples of patients with ARC in Brazil and other tropical countries (3, 4). Blo t 5 is the major allergen of *Blomia tropicalis* and shares 43% of sequence homology with Der p 5 (5) but low to moderate IgE-cross-reactivity between them is reported (6). Sensitivity to house dust mites detected by skin prick tests or serum specific IgE in ARC is frequent but it may not always reflect clinical allergy (7).

The epidemiology of allergic sensitization was assessed in atopic children and adolescents in Curitiba, Southern Brazil. Skin prick tests to *Blomia tropicalis* were positive in 70.7% of patients with asthma and rhinitis (8).

The ISAAC questionnaire and a previously validated allergic conjunctivitis questionnaire have been applied to 4.520 adolescents. Seven hundred (15.5%) had allergic conjunctivitis and females had a higher prevalence of allergic rhinoconjunctivitis and allergic conjunctivitis when compared to males. There was an opposite allergic sensitization pattern with more IgE sensitized boys than girls. Skin prick tests performed in 472 have shown reactions to Bt in 67% of boys and 48% of girls, respectively (9).

Conjunctival provocation test (CPT) is an investigational tool to assess IgE hypersensitivity on the external ocular surface after the topical application of an allergen in an assumed sensitized subject. It is recognized as the only method to confirm or identify which allergen triggers the signs and symptoms of allergic conjunctivitis. CPT is particularly useful for the etiological diagnosis in persistent allergic conjunctivitis, in multisensitized patients and when sensitization is not concordant with the medical history (10).

The purpose of this study was to demonstrate the role of CPT in the diagnosis of allergic conjunctivitis and that *Blomia tropicalis* is a clinical relevant allergen.

## Materials and Methods

### Patient Population

Twenty-six patients (age range 12–48 years) with symptoms of allergic rhinoconjuntivitis for more than 1 year and Bt-sensitized (positive skin prick tests) were included in the study. They were recruited from the outpatient Allergy Clinic, Hospital de Clínicas, Federal University of Paraná (Brazil). Exclusion criteria included pregnant women and subjects with current active conjunctivitis and/or rhinitis, past or current history of other ophthalmic diseases, active eczema, dermatographism or skin lesions in the areas of the skin tests and patients with unstable asthma. Twenty-nine subjects (age range 13–50 years) without a history of ocular and/or nasal allergic symptoms who tested negative to Bt and other common inhalant allergens by SPT served as the control group. Conjunctival provocation tests (CPT) with Bt were performed in all participants of both groups.

### Skin Prick Tests (SPT)

SPT were conducted with standardized extracts of Bt at a concentration of 10 HEP (Alk Abelló—provided by FDA Allergenic, Rio de Janeiro, Brazil) and other common inhalant allergens. Histamine base (5 mg/mL) was used as positive control and diluent (50% glycerin) as negative control. Reactions were graded 15 min later and considered positive if the mean wheal diameter was equal to or >3 mm after the subtraction of wheal diameter of negative control.

### Allergen Conjunctival Provocation Test (CPT)

CPT was carried out using progressive doses of allergen solutions as described by Abelson et al. (11). Bt solutions in normal saline at a serial two-fold dilution were prepared daily at room temperature just before each test. Bt allergen extract 10 HEP for CPT had 462.5 ng/mL of Blo t 5 in 1:1 solution as determined by ELISA assay at Indoor Biotechnologies, Charlottesville, USA.

With a pipette, 20 μL of increasing concentrations (1:32, 1:16, 1:8, 1:4, 1:2) of the extract was instilled in the inferior-external quadrant of the bulbar conjunctiva in the right eye every 20 min until a positive reaction occurred and the test was interrupted. The left eye was used as control and received one drop of saline (NaCl 0.9%) initially and then was challenged with a serial two-fold solutions of the diluent (phenol 0.4% and glycerine 50%) in normal saline the same way done with Blomia solutions. A scoring system of severity was used for each ocular symptom. Itching intensity was rated by the patient according to a 0-4-point scale (0 = absent, 1 = intermittent, 2 = permanent awareness but without desire to rub the eye, 3 = permanent awareness but with desire to rub the eye, 4 = the subject insists on rubbing the eye). The other ocular signs were rated by the investigator as following: redness (0 = absent, 1 = localized within some quadrant, 2 = marked or diffuse reddening in the quadrants, 3 = very marked and diffuse reddening in the quadrants), tearing (0 = absent, 1 = slightly wet eye, 2 = some tears, 3 = profuse tearing, tears roll down the face) and chemosis (0 = absent,1 = detectable with slit lamp, conjunctiva raised from sclera, 2 = visually evident raised conjunctiva in limbal area, 3 = ballooning of conjunctiva). The total ocular symptom score (TOSS) was the sum of each individual symptom score. CPT was considered positive when TOSS was ≥ 5, with both redness and itching scores ≥ 2, respectively. TOSS was rated before and 15 min after the instillation of each allergen dose. Patients should be asymptomatic and off any ocular/nasal and systemic antihistamines and corticosteroids for at least 30 days prior to CPT. All provocation tests were conducted outside the grass pollen season.

A second Bt-conjunctival challenge with the concentration that triggered a positive reaction was performed 1 week later to assess CPT's reproducibility.

This protocol was approved by the local Ethics Committee and signed informed consent was obtained from all participants.

### Statistical Analysis

The data is presented as numbers and percentages. X^2^ test with continuity correction was applied to compare the proportion of SPT and CPT results in both groups. *P* < 0.05 was considered statistically significant.

## Results

Clinical and demographic characteristics of Bt-sensitized and control groups are shown in Table 1. Females predominated significantly over males in the non-allergic participants.

**Table 1:** Demographic and clinical characteristics of the study population.

	**Allergic**	**Non-allergic**	***P*-values**
*n*	26	29	
Age (years)	12–50	13–50	
Median	25 ± 8.5	34 ± 10.6	*p* < 0.42
Gender (*n*)			
Female/Male	14/12	25/4	*p* < 0.0083
Rhinitis and/or CA	23 (88%)	3 (10%)	
Asthma	1 (4%)	3 (10%)	

*CA, allergic conjunctivitis*.

### SPT Results

Of the 26 allergic subjects, two were monosensitized to Bt, 15 had positive SPT to Bt and *Dermatophagoides pteronyssinus* (Dp), eight had positive SPT to Bt, Dp and *Lolium perenne* (Lp) and one reacted to Bt and Lp. SPT were negative to the allergens tested in the 29 controls.

### CPT Results

Bt induced ocular and periocular itching within the first minute (median 3.5 ± 1.2 min) of the allergen exposure, reached a peak in 10–15 min and began to fade after 20 min. Conjunctival hyperemia was observed during the first minute (median 6.2 ± 1.6 min) with a peak at 15–20 min. Itching was present in 92% of positive CPT (*p* < 0.0001).

**Figure 1:**
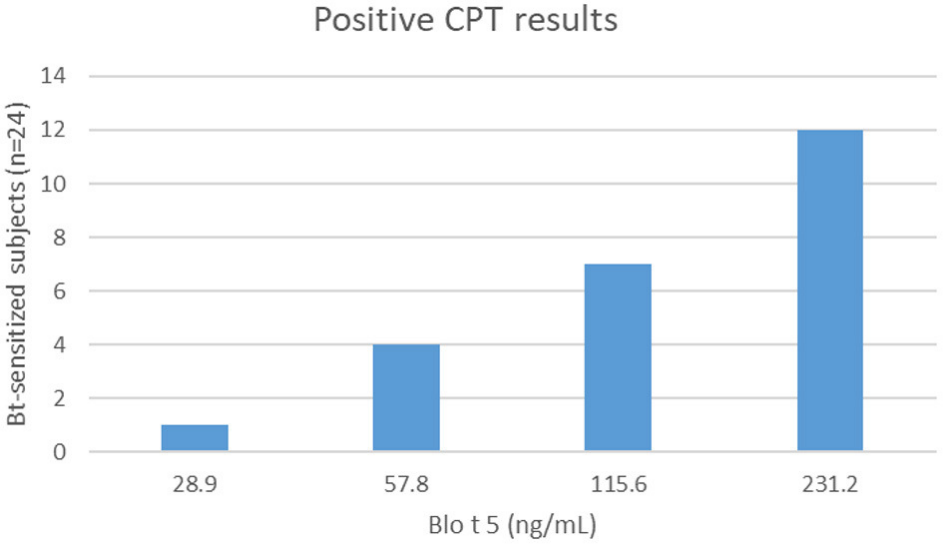
Respondents to conjunctival provocation tests (CPT) and concentrations of Blo t 5.

Most of the patients sensitized to Bt (24/26) reacted to CPT. The dose responses to Blo t 5 for positive challenges varied from 28.9 to 231.2 ng/mL (Figure 1). One Bt-monosensitized subject had a positive conjunctival reaction to Bt and another did not react. No positive CPT was observed in controls. Positive SPT with Bt was significantly predictive of positive CPT (*p* < 0.0001). Degree of sensitization (mean SPT wheal diameter with Bt allergenic extract) was not correlated with concentration of Blo t 5 to elicit a conjunctival reaction. CPT had 92% sensitivity and 100% specificity for diagnosis when compared to SPT results. CPT induced a positive reaction 1 week later with the same allergen dose in 12/22 subjects and with an immediately higher dose in 9/22. One subject who had an initial negative CPT, did not react to a second challenge.

### Adverse Events

Mild transient nasal symptoms (21/24) were the main secondary outcome of positive CPT followed by moderate periorbital edema in 41% (10/24) of the challenges. One controlled asthmatic Bt-sensitized subject developed wheezing and dyspnea during a positive conjunctival challenge with Bt that cleared with inhaled albuterol (400 mcg). There were no reactions to CPT in non-allergic subjects.

## Discussion

This study showed a high rate (92%) of positive CPT in subjects with allergic rhinoconjunctivitis sensitized to Bt and demonstrated that Bt is a causal agent of ocular symptoms. Positive SPT reactions to Bt were highly predictive of positive reactions in the eye (*p* < 0.0001). No positive ocular challenge reactions were observed in the non-sensitized control group. Bt-ocular challenge studies are scarce but similar findings have been described. GarciaRobaína et al. (12) performed a series of conjunctival and bronchial challenges with *Blomia tropicalis* in individuals sensitized to Bt and Dp by SPT or serum specific IgE (s-IgE). There were 62.5% (20/32) of positive CPT with Bt in 18 sensitized subjects and in 2 non-sensitized subjects. Bronchial challenges were positive in 81.8% (9/11) of Bt-sensitized asthmatics. All Dp-sensitized subjects reacted positively to conjunctival and bronchial challenges with Dp except one who was sensitized only to Bt and did not react to the bronchial provocation test. In general, challenges were positive when SPT and/or s-IgE tests were positive but individuals who were sensitized to different mite species might only react to one of them.

Reactions to glycerin and preservatives could account for irritant effect on the ocular surface. In our study, all the procedures were conducted in a control group of asymptomatic non-allergic subjects and none of them reacted to the solutions tested. Furthermore, CPT repeated 1 week later induced reactions with the same allergen dose or with an immediately higher dose. The reproducibility of the tests could minimize an irritant effect of preservatives of the extract.

Stanaland et al. (13) demonstrated responses to nasal challenge to Bt in 83% of Bt-sensitized subjects. In their region, Bt was found in 33% of house dust samples in concentrations >150 mites per gram of dust (14) and sensitization to Bt detected by SPT accounted for 38% of allergic respiratory symptoms (15). No positive nasal challenge reaction to Bt was found in the group of individuals sensitized to other species of house dust mites such as *Dermatophagoides pteronyssinus* and *Dermatophagoides farinae*. Nevertheless, Bt was allergenic and should be considered as a cause of allergic rhinitis.

In Brazil, Barreto et al. (16) demonstrated the allergenicity of Bt by nasal challenges (NPT) in children with perennial allergic rhinitis who were sensitized to Bt and Dp. Specific and non-specific nasal mucosa reactivity were assessed. There were 60% of positive challenges to Bt and 90% to Dp. Eight out of 10 histamine NPT were positive showing a high prevalence of non-specific hyperreactivity of the nasal mucosa in children with allergic rhinitis. Conjunctival hyperreactivity to non-specific stimuli has also been documented in allergic and non-allergic patients (17). CPT with hyperosmolar solutions in patients with ocular symptoms have elicited conjunctival hyperemia, mild itching/burning and tearing in 84% of allergic patients sensitized to dust mites and grass but 16% of non-allergic subjects also had positive ocular challenges to glucose solutions (18). Allergic subjects exhibit more conjunctival responsiveness than non-allergic subjects, even when asymptomatic, probably due to a minimal persistent inflammation process. We could speculate that the perennial exposure to house dust mites might be a factor that could contribute to conjunctival inflammation and hyperreactivity.

The frequency of sensitization to *Blomia tropicalis, Dermatophagoides pteronyssinus* and *Dermatophagoides farinae* in asthmatics from different cities in Latin America have been reported between 60 and 97%. *Blomia tropicalis* is considered a common sensitizer in Brazilian atopic children (19). In patients with atopic dermatitis, having sensitization to rBlo t 5 is highly specific and sensitive although there was also high sensitization to the components nDer p 1/n Der f 1 in severe forms of atopic dermatitis (20).

In general, CPT with Bt was a safe procedure triggering self-limited ocular and nasal symptoms mainly related to the early phase reaction of Ige-allergic inflammation. Only one asymptomatic asthmatic patient developed mild wheezing during CPT. A study realized in Singapore with nasal challenges with Bt provoked late-phase reaction wheezing in patients with allergic rhinitis and a history of asthma (21). Even though most adverse reactions of CPT are mild, there is potential for more severe/systemic responses and it should preferably be performed in centers where side effects can be handled (22).

Hypersensitivity to *Blomia tropicalis* is usually based on the results of SPT or serum levels of specific IgE to the whole allergen or to Blo t 5, its major allergen (23–25). Despite the frequent sensitization observed in SPT and S-IgE, Blo t 5 concentration in house dust samples in Brazil (26) and in other tropical climate countries (27) have been found to be low with predominance of Dp or other mite species. Another source of discrepancy in this issue could be the existence of different regional variants or isoforms of Blo t 5 that could be underdetected by the ELISA monoclonal assays. Relative abundance, instability of Blo t 5 or reliability of the assay used may account for these findings.

One limitation of this study was that standardization was solely based on Blo t 5 allergen content, other Bt allergens have been reported to contribute to the allergenic activity of Bt extracts and usually Blo t 5 is only a minor fraction of the protein content in these products (3, 23).

The small number of subjects and the predominance of females in the control group could both be confounding factors. Target organ challenge studies may demonstrate a specific allergen as the sensitizer and the trigger of symptoms (28). In our study, Bt-extract was standardized and the concentration of Blo t 5 that elicited signs and symptoms of allergic conjunctivitis was known. The results are strengthened by the biological effect of the extract and the dose-response behavior, although it could be misleading to attribute all or most effect solely to Blo t 5. It is essential to use standardized allergens for conjunctival challenges to obtain accurate and reproducible responses of true sensitization (29). From a clinical perspective, CPT could be useful to select allergen extracts for SPT and immunotherapy.

## Conclusion

This study demonstrated that Bt may cause allergic conjunctivitis in our population. In addition, CPT is a safe and reproducible test if standardized allergens are used. SPT is an indicator of clinical relevance of sensitization in patients with allergic conjunctivitis.

## Data Availability Statement

The raw data supporting the conclusions of this article will be made available by the authors, without undue reservation.

## Ethics Statement

The studies involving human participants were reviewed and approved by Comite de Etica em Pesquisa em Seres Humanos Hospital de Clinicas–UFPR. Written informed consent to participate in this study was provided by the participants' legal guardian/next of kin.

## Author Contributions

All authors listed have made a substantial, direct and intellectual contribution to the work, and approved it for publication.

## Conflict of Interest

The authors declare that the research was conducted in the absence of any commercial or financial relationships that could be construed as a potential conflict of interest. The handling editor declared a past co-authorship with one of the authors NR.
